# Successful Treatment of Respiratory Syncytial Virus Infection in an Immunocompromised Patient With Ribavirin

**DOI:** 10.7759/cureus.16930

**Published:** 2021-08-06

**Authors:** Wasey Ali Yadullahi Mir, Dhan B Shrestha, Wajahath Rana, Shravani Reddy Yelma Reddy, Ayusha Paudel, Larissa Verda

**Affiliations:** 1 Department of Internal Medicine, Mount Sinai Hospital, Chicago, USA; 2 Department of Infectious Diseases, Mount Sinai Hospital, Chicago, USA; 3 Department of Internal Medicine, SRM Medical College Hospital and Research Centre, Chennai, IND; 4 Department of Emergency Medicine, Alka Hospital Pvt Ltd, Kathmandu, NPL

**Keywords:** respiratory syncytial virus, ribavirin, leukemia, respiratory tract infection, elderly

## Abstract

Respiratory syncytial virus (RSV) is a frequent cause of respiratory tract infections in children. Still, it can also cause seasonal outbreaks affecting persons of all ages, especially those with comorbidities or immunocompromised states. Ribavirin is one of the two approved therapies for the treatment of RSV respiratory tract infections. Unfortunately, its aerosolized formulation has been approved only in children, and the oral formulation is not frequently used to treat the infection. However, ribavirin has demonstrated morbidity and mortality benefit in immunocompromised patients. A 70-year-old female had started chemotherapy for a diagnosis of B-cell acute lymphoblastic leukemia (B-ALL). She developed an upper respiratory tract infection (URTI) along with positive RSV status. We started her on oral ribavirin therapy, which had to be stopped after five days of treatment due to an acute hemolytic reaction. She was re-initiated on oral ribavirin after cessation of medication for seven days and showed improvement. Therefore, ribavirin can be used in immunocompromised patients with RSV infection under proper supervision.

## Introduction

Respiratory syncytial virus (RSV) is a single-stranded, negative-sense, enveloped RNA virus that commonly infects children worldwide [1]. It has now been recognized as a problem in the adult population, with significant mortality and morbidity in immunocompromised hosts [2]. The clinical manifestations of RSV infection in an immunocompromised individual depends on the degree of immunosuppression [2]. Ribavirin use has demonstrated effectiveness in preventing the progression of RSV infection from lowering respiratory tract infection [3]. Generally, inhaled ribavirin is used to treat RSV infection; however, we present a patient with newly diagnosed B-cell acute lymphoblastic leukemia (B-ALL) with RSV infection, which significantly benefited with oral ribavirin therapy.

## Case presentation

A 70-year-old female with a past medical history of hypertension and hyperlipidemia presented with a history of gastroesophageal reflux disease. She was taking amlodipine, pantoprazole, and atorvastatin. On admission, she presented with fatigue and weakness. The patient was afebrile and hemodynamically stable. Physical examination findings were unremarkable. Chest examination was normal, with no rales or wheezes. Cardiac and abdominal examinations were unremarkable. The patient had pancytopenia with hemoglobin of 11.8 gm%, platelet count of 87000 per microlitre, and leukocyte count of 2200 per microlitre. The uric acid level was elevated to 6.8 mg/dl.

A bone marrow biopsy was performed, which was positive for B-ALL. The patient was diagnosed with B-ALL, for which she was started on chemotherapeutic drugs. When on chemotherapy, she experienced generalized weakness, fatigue, and decreased appetite for three months. She also developed upper respiratory tract symptoms. The respiratory viral panel was sent, and it turned out to be positive for RSV the next day, and oral ribavirin was started immediately. The initial chest radiograph was negative for acute cardiopulmonary findings.

As the patient was immunocompromised with an RSV positive status, she was started on oral ribavirin 15-20 mg/kg of body weight in three divided doses per day for 10 days. During the treatment course, hemoglobin dropped to 6.7gm% on the fifth day of treatment. As a result, oral ribavirin was held. Following the discontinuation of the ribavirin and completing a round of chemotherapy, she became neutropenic with a temperature of 101 °F. She also developed hypoxemic respiratory failure with diffuse dense bilateral patchy infiltrates requiring intubation.

She was promptly started on empiric and prophylactic antibiotics for immunosuppression. Blood cultures, sputum culture, serology for Histoplasma and Legionella, and bronchoscopy sample for pneumocystis were sent and found to be negative. However, a repeat respiratory panel was positive for RSV. After seven days without ribavirin, she was restarted on a new 10-day course of ribavirin. The patient showed recovery over few days and was extubated. She was discharged from the hospital following her recovery.

## Discussion

Currently, no specific guidelines are available for the treatment of RSV in the adult population [4]. Ribavirin use has shown reduced mortality in RSV infection of adults [5]. It is available in three formulations: aerosolized, oral, and intravenous, out of which intravenous formulation is not approved by the Food and Drug Administration (FDA) [5]. FDA has approved the use of aerosolized ribavirin in infants and young children but not in adults [4,6]. Despite not being approved for adults, aerosolized ribavirin is predominantly used to treat RSV in adults [7,8]. However, a handful of studies have shown the benefit of oral ribavirin therapy [9-11].

Aerosolized ribavirin should be administered via a scavenging tent as it is teratogenic [9]. It is also associated with adverse effects like bronchospasm, which is undesirable in patients with RSV infection, nausea, and anemia [12]. It should be administered for six to eighteen hours daily, which is cumbersome [9]. Oral ribavirin is largely devoid of respiratory symptoms, with hemolytic anemia and gastrointestinal upset being the major side effects [13].

The cost of aerosolized and oral ribavirin therapy is also drastically different. For example, a single-day course of aerosolized ribavirin ran upwards of $11581, while oral therapy was about $22 in 2016 [12]. Therefore, the economic benefit during the choice of formulation when initiating therapy cannot be overlooked.

Due to the economic benefit and better respiratory symptoms profile, oral ribavirin therapy has started gaining prominence in clinical usage. In a small cohort of patients receiving oral ribavirin therapy at the University of California, San Francisco Medical Center, the use of oral ribavirin resulted in reduced expenditure without a significant increase in worse outcomes [12]. Another study reviewing the patients’ outcome receiving oral ribavirin versus aerosolized formulation at the University of Texas MD Anderson Cancer Center showed no increased efficacy of aerosolized ribavirin. Therefore, it postulated that oral ribavirin might be a safe and effective alternative to the aerosolized formulation [5].

Patients receiving oral therapy experienced fewer side effects along with ease of administration. Therefore oral administration of ribavirin in adults can be considered in limited circumstances, as in this case [12].

## Conclusions

RSV infection in an immunocompromised individual frequently results in lower respiratory tract infection. Ribavirin has demonstrated benefit in the morbidity and mortality of such patients. Although aerosolized ribavirin has been used for treatment purposes, oral formulation is gaining prominence in use due to its better safety profile and economic benefit. We report a case of successful treatment of RSV infection in an immunocompromised individual with the oral formulation of ribavirin.
